# The association between body fat and musculoskeletal pain: a systematic review and meta-analysis

**DOI:** 10.1186/s12891-018-2137-0

**Published:** 2018-07-18

**Authors:** Tom P. Walsh, John B. Arnold, Angela M. Evans, Alison Yaxley, Raechel A. Damarell, E. Michael Shanahan

**Affiliations:** 1 College of Medicine and Public Health, Flinders University, Bedford Park, South Australia 5042 Australia; 2 Department of Orthopaedics and Trauma, The Queen Elizabeth Hospital, Woodville South, South Australia 5011 Australia; 3 Alliance for Research in Exercise, Nutrition and Activity, Sansom Institute for Health Research, School of Health Sciences, University of South Australia, Adelaide, South Australia 5000 Australia; 4 Discipline of Podiatry, College of Science, Health and Engineering, La Trobe University, Bundoora, VIC 3086 Australia; 5 Nutrition & Dietetics, College of Nursing and Health Sciences, Flinders University, Bedford Park, South Australia 5042 Australia; 6 Department of Rheumatology, Southern Adelaide Local Health Network, Bedford Park, South Australia 5042 Australia

**Keywords:** Obesity, Body composition, Musculoskeletal, Pain, Adiposity

## Abstract

**Background:**

Obesity and musculoskeletal pain are strongly related, but there is emerging evidence that body fat, not body weight, may be a better indicator of risk. There is, therefore, a need to determine if body fat is associated with musculoskeletal pain as it may improve management strategies. The aim of this systematic review was to investigate the association between body fat and musculoskeletal pain.

**Methods:**

Seven electronic databases were searched from inception to 8th January 2018. Cross-sectional and longitudinal studies investigating the association between measures of body fat and musculoskeletal pain were included. All included articles were assessed for methodological rigour using the Epidemiology Appraisal Instrument. Standardised mean differences (SMDs) and effect estimates were pooled for meta-analysis.

**Results:**

A total of 10,221 citations were identified through the database searching, which after abstract and full-text review, yielded 28 unique articles. Fourteen studies were included in the meta-analyses, which found significant cross-sectional associations between total body fat mass and widespread pain (SMD 0.49, 95% CI 0.37–0.61, *p* < 0.001). Individuals with low-back pain and knee pain had a higher body fat percentage than asymptomatic controls (SMD 0.34, 95% CI 0.17–0.52, p < 0.001 and SMD 0.18, 95% CI 0.05–0.32, *p* = 0.009, respectively). Fat mass index was significantly, albeit weakly, associated with foot pain (SMD 0.05, 95% CI 0.03–0.06, p < 0.001). Longitudinal studies (*n* = 8) were unsuitable for meta-analysis, but were largely indicative of elevated body fat increasing the risk of incident and worsening joint pain. There was conflicting evidence for an association between body fat percentage and incident low-back pain (3 studies, follow-up 4–20 years). Increasing knee pain (1 study) and incident foot pain (2 studies) were positively associated with body fat percentage and fat mass index. The percentage of items in the EAI graded as ‘yes’ for each study ranged from 23 to 85%, indicating variable methodological quality of the included studies.

**Conclusions:**

This systematic review and meta-analysis identified positive cross-sectional associations between increased body fat and widespread and single-site joint pain in the low-back, knee and foot. Longitudinal studies suggest elevated body fat may infer increased risk of incident and worsening joint pain, although further high-quality studies are required.

**Electronic supplementary material:**

The online version of this article (10.1186/s12891-018-2137-0) contains supplementary material, which is available to authorized users.

## Background

Musculoskeletal conditions, manifesting as pain in soft tissues and joints, are a leading cause of disability [1]. Worldwide, they are second only to mental and behavioral problems in contributing to the total years lived with disability [2]. Musculoskeletal pain can lead to an avoidance of physical activity [3] and weight gain [4]. Excessive weight gain may result in the development of obesity and there is a strong bidirectional relationship between obesity and musculoskeletal pain [5], but understanding how excessive body weight and pain are related is important as it guides therapy.

The implication that excessive loading of joints is directly related to pain likely oversimplifies the complex relationship between obesity and pain. This is demonstrated by an abundance of studies with often conflicting findings regarding the nature of the relationship between mechanical loading and pain [6–10]. Moreover, whilst ground impact forces are positively related to obesity, lean mass (i.e. muscle) is negatively associated with impact force and may be protective, suggesting that body tissues should not all be considered homogeneous [11].

Obesity is commonly defined as ≥30 kg/m^2^ on the body mass index (BMI) scale, which is calculated by dividing body weight (kg) by body height (m) squared. This scale, however, treats all body tissue as homogeneous and it does not account for either the type or the distribution of body weight [12]. The BMI is not a good measure of adiposity (body fatness) as it does not account for age or gender differences [13]. Furthermore, given the association between BMI-defined obesity and musculoskeletal pain extends to both weight-bearing [14] and non-weight-bearing joints [15], it follows that the mechanism underpinning this relationship may extend beyond excessive mechanical loading alone, which is implied with the BMI. Fat mass index (FMI) is a more relevant measure in having or predicting pain [16], suggesting the type of tissue is important. It is also now well-recognised that adipose tissue is an active endocrine organ that secretes many active cytokines and hormones [17], some of which may be related to the development of musculoskeletal pain.

Recent cross-sectional and longitudinal studies are beginning to highlight the important role of body composition in the development and worsening of joint pain [18–20]. Body composition can be analysed using a number of techniques including dual energy x-ray absorptiometry, bioelectrical impedance analysis and skin-fold thickness, although this method has challenges with increasing levels of obesity [21]. Whilst much attention is directed toward the strong association between BMI-defined obesity and musculoskeletal pain, there are metabolic [22, 23], structural [24] and psychological mechanisms [25] that may link adiposity and pain. There is, therefore, a need to determine whether body fat is associated with musculoskeletal pain as this understanding may improve management strategies. The aim of this systematic review was therefore to investigate the association between body fat and musculoskeletal pain.

## Methods

This systematic review was conducted in accordance with the Preferred Reporting Items for Systematic Reviews and Meta-Analyses (PRISMA) statement guidelines [26]. This systematic review was registered at the International Prospective Register of Systematic Reviews (PROSPERO) on 12th August 2017 (http://www.crd.york.ac.uk/PROSPERO/), registration number: CRD42017074289.

### Search strategy for identification of studies

The following databases were searched on 9th August 2017: Medline (Ovid); PubMed (non-Medline content only); Embase (OVID); Scopus; CINAHL (EBSCOhost); Cochrane Central Register of Controlled Trials; and Web of Science. All databases were searched from inception to current date. Reference lists from suitable papers were also investigated and included prior to applying exclusion and inclusion criteria. Broad MeSH terms and keywords were used, combining musculoskeletal pain and body composition. The search terms were broad to ensure capture of all relevant studies. Additional file 1 illustrates the full search strategy used for this systematic review, and minor modifications to search terms were required depending on the database searched. Database searching and registration for automatic e-alerts were also continued until the review was finalised (8th January 2018).

Following removal of duplicates, two reviewers (TPW and JBA) applied the predetermined selection criteria to all articles by reading the title and abstract alone. Where discrepancies between article selections existed, the reviewers discussed these discrepancies to form a consensus, a third reviewer was not required to arbitrate a consensus for this review. Articles were then assessed for eligibility by full-text review.

### Eligibility criteria

Articles from English language, peer-reviewed, scientific journals were eligible for inclusion in this review if they reported studies that examined the association between body composition and musculoskeletal pain. Studies were included if all participants were aged at least 18 years, had musculoskeletal pain recorded via self-report or questionnaire (or were controls) and had an assessment of body fat. Studies specifically investigating participants with inflammatory conditions or autoimmune diseases were excluded. Further exclusion criteria were; unclear assessment of musculoskeletal pain or body composition, letters to the editor and editorials, opinion pieces and non-English language publications.

### Assessment of methodological quality

All included articles were assessed for methodological rigour using the Epidemiology Appraisal Instrument (EAI) [27]. This tool has been shown to demonstrate good reliability and content validity [28]. A number of items from the EAI were omitted as they were not applicable to non-interventional studies (Questions 10,12,20,22-24,35,37,40) as per previous reviews of observational studies investigating musculoskeletal disorders [29, 30]. The covariates considered important for questions 11 and 36 were age, gender and a measure of psychological health. As it is not known if each question of the EAI is equally weighted, rather than providing a quality assessment score for each study, a summary score for each question is reported. A summary (%) of the number of questions a study scored ‘yes’ on is also reported.

### Data extraction and analysis

To reduce the risk of bias, author and publication details were removed prior to data extraction. Where available the relevant data (means, medians, standard deviations (SDs), odds ratios (ORs), relative risks (RRs), confidence intervals (CIs) and *p* values) were recorded for each study. Where available, multivariable OR (95% CI) were extracted in preference to unadjusted OR (95% CI). For studies reporting means and standard deviations, effect sizes (Cohen’s *d*) and CIs were calculated. According to Cohen [31], effect sizes were interpreted as 0.2, small; 0.5, medium; and 0.8, large. Widespread pain was defined as ≥5 painful joints, which is modeled on the criteria of the American College of Rheumatology [32]. Multi-site pain was defined as > 1 but < 5 painful joints. For those studies investigating multi-site or widespread pain, the differences were calculated between the no pain group and the multi-site / widespread pain group. Meta-analysis was performed where more than one study reported on the same parameter, grouped by widespread or single-site pain location. Only the gender-specific sample size was used when entering gender-stratified data into the meta-analysis.

The OR and CIs, and SMD (Cohen’s *d*) were pooled for meta-analysis by the standard approach, weighted by the inverse variance method. Odds ratios and CIs were converted to SMDs for meta-analysis [33]. Statistical heterogeneity was assessed for each site using the *I*^*2*^ statistic. Potential publication bias was assessed graphically using a funnel plot [34] and Egger’s regression intercept for low-back pain, knee pain and foot pain. Both heterogeneity and publication bias were considered, accepting the fact that the power was low because of the small number of studies for each site. Sensitivity analysis was performed via the one-study removed test (removal of individual studies out of the model in turn), which gauges each study’s impact on the overall pooled effect size. A *p*-value less than 0.05 (two-tailed) was considered statistically significant. All analyses were conducted using Comprehensive Meta Analysis v3.0 (Biostat, NJ, USA).

## Results

**Fig. 1:**
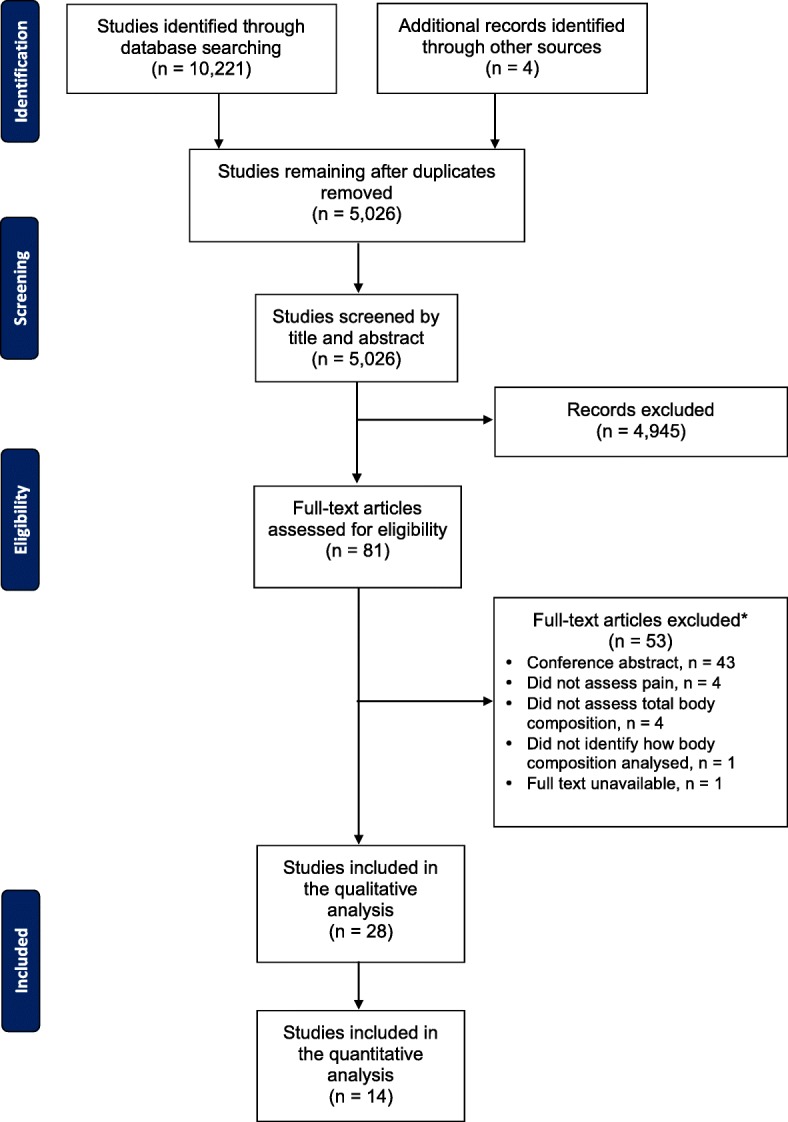
Selection process for inclusion of articles in this review of the association between body fat and musculoskeletal pain

The initial literature search yielded a total of 10,221 citations, which was reduced to 5026 following the removal of duplicates. These 5026 articles were screened based on their title and abstract, where a further 4945 articles were excluded, leaving 81 articles that underwent full-text review. After 53 articles were excluded, 28 unique articles were included in this review [16, 18–20, 35–58]. Twenty-two articles reported cross-sectional data [16, 18, 20, 35–53] and eight articles [16, 19, 35, 54–58] provided longitudinal data (two articles reported both cross-sectional and longitudinal data [16, 35]). Four articles used participants from the same study [18–20, 40], and there were three other instances of articles using data, reporting different outcomes, from the same study [47, 58, 35, 39, 55] and [38, 42], leaving 21 unique studies. The regions with multiple studies using the same parameter were widespread, low-back, knee and foot and thus these were included in the quantitative analysis (*n* = 14) [16, 35–37, 39–43, 47–49, 51, 52]. All of the studies included in the meta-analysis were cross-sectional. There were fewer longitudinal studies, with most studies only investigating one site (other than low-back) with variable follow up time, therefore these data did not undergo meta-analysis. Details of study selection have been recorded (Fig. 1) following the guidelines set by PRISMA.

### Study characteristics

A variety of sites for musculoskeletal pain were investigated, including the neck, low-back, knee and foot. The low-back was the most common region investigated, with 15 articles including this site in their analysis [18, 20, 35–38, 43, 46–48, 51, 52, 56–58]. Three articles investigated the association between multi-site / widespread pain and body composition [18, 35, 36], while another investigated multiple regions, but stratified the analysis by these regions [37]. One study [54] used body composition as a predictor for any injury and thus a specific region was not investigated. Body composition was analysed with dual energy x-ray absorptiometry in 13 articles [16, 18–20, 35, 36, 38–43, 55], bioelectrical impedance analysis in 10 articles [37, 44–50, 56, 57], and skin fold thickness in 5 articles [51–54, 58]. Body composition was generally reported as a percentage of body fat (17/28 articles) [37, 39, 43–52, 54–58]. The cross-sectional articles consisted of; population-based (*n* = 7), clinic-based (*n* = 7), musculoskeletal pain (*n* = 5), occupational-based (*n* = 2) and unknown (*n* = 1). The longitudinal articles were largely population-based (*n* = 6) along with occupational-based (n = 1) and military-based (n = 1). The longitudinal articles varied in follow-up from 3 months [54] to > 20 years [58], but most were between 3 and 5 years.

### Participant characteristics

The studies included in this systematic review reported on 12,942 participants, with studies from Asia, Europe, South America and Australia. Both men and women were represented in most studies, although gender-specific studies accounted for > 35% of the total [38, 41, 42, 46, 47, 49, 52, 54, 57, 58]. Mean age in the cross-sectional studies ranged from 20.7 years [50] to 74.4 years [41], while the longitudinal studies ranged from 19.0 years [54] to 64.6 years [16]. Most cross-sectional studies included participants with mean BMIs of < 30 kg/m^2^, however four included participants with a mean BMI of > 30 kg/m^2^ [18, 20, 40, 53]. The mean BMI of the participants from the longitudinal studies ranged from 20.8 kg/m^2^ [54] to 29.6 kg/m^2^ [19].

### Methodological quality assessment

The results of the methodological quality assessment are provided in Additional file 2. The summary scores for each question ranged from 4 to 96%, with 14/34 questions scoring above 50%. The percentage of items in the EAI graded as ‘yes’ for each study ranged from 23 to 85%, indicating variable methodological quality of the included studies. There were common, strong themes among the studies with the clear descriptions of the aims, study design and results reported in most studies (> 85%). There were, however, a number of consistent methodological limitations; the reliability and validity of the instruments used was often under-reported, a sample size calculation was mostly not reported (96%) and the generalisability of the findings was questionable in over 80% of the included studies. Whilst there was adjustment for a number of other variables e.g. smoking, physical activity, self-reported arthritis, adjustment for all of the important confounding variables was reported in less than 30% of the articles [16, 18, 19, 35, 38, 40, 42, 56]. One article considered psychological health alone [44], one article considered age alone [58], six articles considered both age and gender [20, 36, 37, 41, 55, 57]. Only three articles [16, 40, 42] provided data that were adjusted for the important confounding variables (age, gender, psychological health) that were also used in the meta-analyses.

### Meta-analysis

**Fig. 2:**
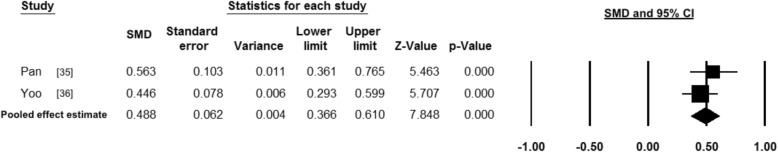
Forest plot of effect sizes and 95% confidence intervals for widespread pain and total body fat relative to controls

**Fig. 3:**
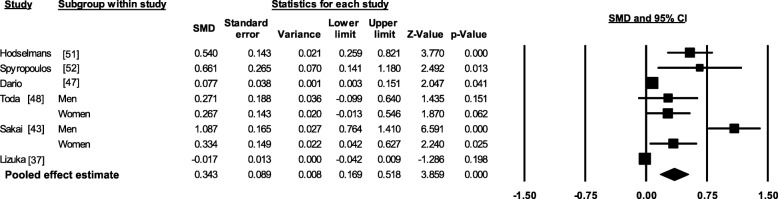
Forest plot of effect sizes and 95% confidence intervals for low-back pain and body fat percentage relative to controls

**Fig. 4:**
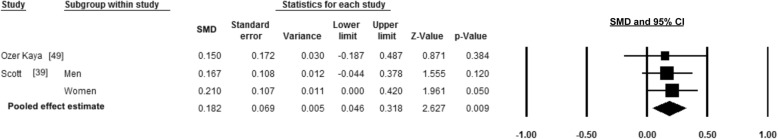
Forest plot of effect sizes and 95% confidence intervals for knee pain and body fat percentage relative to controls

**Fig. 5:**
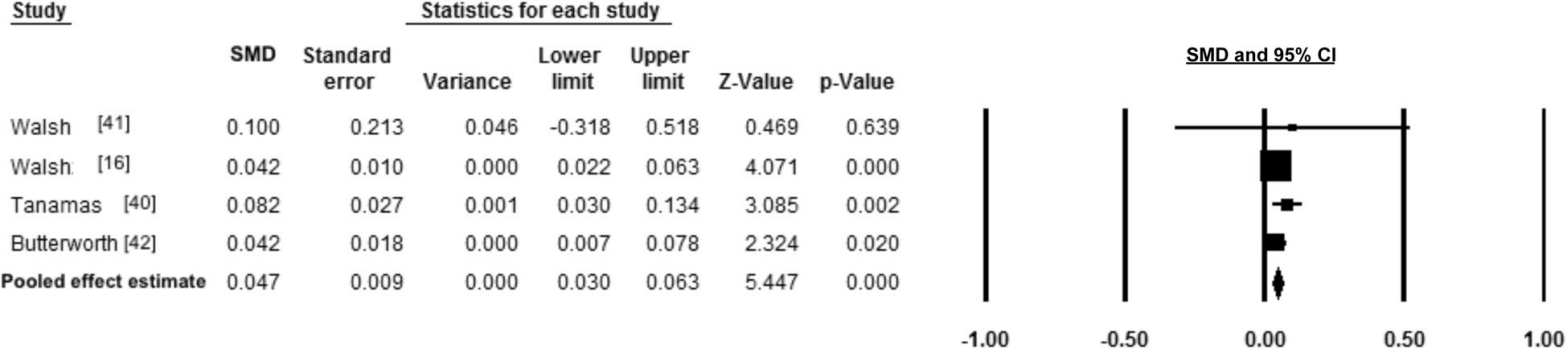
Forest plot of effect sizes and 95% confidence intervals for foot pain and fat mass index relative to controls

Meta-analysis of cross-sectional single-site and widespread pain studies found significant associations between body fat and pain (Figs. 2, 3, 4 and 5), summarised in Table 1. There was a positive medium effect size between total body fat mass and widespread pain (SMD 0.49, 95% CI, 0.37–0.61, *p* < 0.001 and *I*^*2*^ *< 0.001, p* = 0.366). Single-site musculoskeletal pain also had positive associations with body fat. Low-back pain and body fat percentage had a combined small-medium effect size (SMD 0.34, 95% CI 0.17–0.52, *p* < 0.001), but there was a significant level of heterogeneity (*I*^*2*^ *=* 91.21, *p* < 0.001). Body fat percentage and knee pain had a small effect (SMD 0.18 95% CI, 0.05–0.32, *p* = 0.009 and *I*^*2*^ *< 0.001, p* = 0.941), while the pooled FMI and foot pain had a small effect (SMD 0.05, 95% CI, 0.03–0.06, *p* < 0.001 and *I*^*2*^ *< 0.001, p* = 0.564).

**Table 1:** Selected characteristics of the cross-sectional articles included in the review (*n* = 22)^a^

Study / country / reference	Sample size, n	Age, y	BMI, kg/m^2^	Body composition assessment	Parameter investigated	Body composition (Case, control)	Effect size (CI) Cohen’s *d*	OR (CI)	Included in the meta-analyses
Widespread pain (≥ 5 sites)									
Pan, Australia [35] ^d^	336 widespread pain, 137no pain	Baseline 63.3 (7.7) widespread pain, 62.2 (7.2) no pain	Baseline 28.8 (5.3) widespread pain, 26.2 (3.9) no pain	DXA	Total fat mass	30.0 (9.5) kg, 25.0 (7.1) kg	0.56 (0.36–0.76)	N/A	Yes
Yoo, Republic of Korea [36]	229 widespread pain, 618 no pain	60.8 (8.6)	24.3 (3.2)	DXA		19.1 (6.1) kg, 15.9 (7.5) kg	0.45 (0.29–0.60)	N/A	Yes
Multi-site pain (3 sites)									
Brady, Australia [18]	133 (104 women), 42 multi-site pain 27 no pain	47.9 (45.0, 50.7)^c^ multi-site pain 46.3 (42.8, 50.0) no pain	36.6 (34.1, 39.2)^c^ multi-site pain 28.4 (25.2, 31.6) no pain	DXA	FMI	16.2 (14.5–17.9) kg/m^2^ multi-site pain, 11.0 (8.8–13.1) kg/m^2^ no pain	N/A	N/A	No
Temporomandibular joint pain									
Jordani, Brazil [44]	299 (229 women), 159 pain, 70 no pain	37.7 (12.2) pain 35.9 (13.6) no pain	Not stated	BIA	Body fat percentage	N/A	N/A	1.58 (0.72–3.48)	No
Neck pain									
Yalcinkaya, Turkey [45]	160 (80 women), 40 case, 40 control	44.6 (10.2) case 40.8 (8.0) control	28.4 (4.3) case 28.3 (3.9) control	BIA	Body fat percentage	Case Women: 46.6 (9.6) % Men: 37.6 (6.0) % Control Women: 46.0 (9.8) % Men: 34.2 (6.0) %	Women 0.06 (− 0.38–0.50) Men 0.57 (0.11–1.01)	N/A	No
Neck and shoulder pain									
Iizuka, Japan [37]^e^	273 (179 women)	64.3 (13.2)	23.4 (2.9)	BIA	Body fat percentage	N/A	N/A	0.98 (0.94–1.03)	No
Low-back pain									
Hodselmans, Netherlands [51]	101 (47 women)	39.2 (9.6)	Not stated	Skin fold	Body fat percentage	30.4 (8.2) %, 26.4 (6.1) %	0.55 (0.27–0.83)	N/A	Yes
Spyropoulos, Greece [52]	60 (all women), 30 case, 30 control	41.7 (7.3) case 42.2 (7.3) control	27.1 (3.4) case 25.3 (3.1) control	Skin fold		34.7 (5.1) %, 31.3 (5.2) %	0.66 (0.13–1.17)	N/A	Yes
Iizuka, Japan [37]^e^	273 (179 women)	64.3 (13.2)	23.4 (2.9)	BIA		N/A	N/A	0.97 (0.93–1.02)	Yes
Dario, Spain [47]	687 (all women), 313 pain, 374 no pain	53.6 (7.4) pain 52.2 (7.4) no pain	27.7 (5.2) pain 26.8 (4.6) no pain	BIA	Body fat percentage	N/A	N/A	1.15 (1.01–1.32)	Yes
Toda, Japan [48]	330 (206 women), 203 case 127 control	Men: 55.6 (8.8) case, 57.7 (9.8) control Women: 60.0 (9.2) case, 57.6 (8.1) control	Men: 23.9 (2.4) case, 23.9 (3.1) control Women:22.7 (3.4) case, 22.7 (3.3) control	BIA		Women 29.7 (6.8) %, 27.9 (6.7) % Men 23.8 (5.2) %, 22.3 (6.1) %	Women 0.27 (−0.01–0.54) Men 0.27 (− 0.10–0.64)	N/A N/A	Yes
Sakai, Japan [43]	660 (311 women), 100 case, 560 control	74.4 (6.0) case, 73.2 (7.6) control	23.6 (3.2) case, 24.2 (3.5) control	DXA		Women: 41.1 (4.1) %, 34.3 (8.8) % Men: 35.8 (6.7) %, 27.7 (7.6) %	Women: 0.83 (0.09–1.54) Men: 1.08 (0.76–1.40)	N/A N/A	Yes
Celan, Slovenia [46]	112 (all men), 76 pain 36 no pain	44.2 (5.6), range 31–56	27.7 pain, 27.9 no pain	BIA		Case 26.4% Control 25.5%	N/A	N/A	No
Urquhart, Australia [20]	135 (113 women), 29 high pain, 106 no or low pain	47.4 (9.0) range 25–62	32.6 (8.7), range 18–55	DXA	Total fat mass	N/A	N/A	Pain intensity 1.19 (1.04–1.38) Disability 1.41 (1.20–1.67)	No
Chou, Australia [38]	820 (all men), 124 high pain, 696 no or low pain	62.9 (14.0) high pain 58.1 (17.1) no or low pain	28.6 (4.5) high pain 27.2 (4.1) no or low pain	DXA	Total fat mass	High pain disability / intensity 25.9 (7.9) kg No or low disability / intensity 23.0 (8.6) kg	0.34 (0.15–0.53)	N/A	No
Knee pain									
Ozer Kaya, Turkey [49]	149 (all women), 52 cases, 97 controls	42.6 (4.1) case 41.7 (4.2) control	30.5 (5.3) case 29.4 (4.6) control	BIA	Body fat percentage	39.3 (7.9) %, 38.1 (7.7) %	0.15 (−0.19–0.49)	N/A	Yes
Scott, Australia [39]	709 (357 women), 311 pain, 398 no pain	Men: 62.0 (7.2) pain, 63 (7.3) no pain Women: 61.7 (7.5) pain, 62.0 (7.0) no pain	Men: 28.2 (3.8) pain 27.0 (3.5) no pain Women: 28.2 (5.6) pain, 27.0 (4.4) no pain	DXA		Women 40.1 (5.5%, 39.0 (5.0) % Men 28.0 (5.2) %, 27.2 (4.4) %	Women 0.21 (0.00–0.42) Men 0.17 (− 0.04–0.38)	N/A N/A	Yes
Sutbeyaz, Turkey [53]	56 (32 women), 28 cases 28 control	44.0 (10.2) case 43.7 (10.0) control	33.3 (3.7) case 34.8 (3.5) control	Skin fold	Total fat mass	Case 29.4 (7.2) kg Control 33.6 (7.5) kg	−0.57 (−1.10- -0.03)	No	No
Shin pain									
Sabeti, Iran [50]	35 (gender not stated), 17 cases 18 control	21.1 (2.3) case 20.7 (2.5) control	21.7 (2.7) case 20.7 (2.2) control	BIA	Body fat percentage	Case 27.8 (7.2) % Control 23.4 (5.8) %	0.68 (−0.02–1.34)	N/A	No
Foot pain									
Walsh, Australia [41]	88 (all women), 44 cases, 44 control	56.6 (10.3) case 56.7 (6.5) control	29.3 (9.9) case 27.6 (10.5) control	DXA		12.5 (5.1) kg/m^2^, 12.0 (4.9) kg/m^2^	0.10 (−0.32–0.52)	N/A	Yes
Walsh, Australia [16]	1066	64.6 (10.3)	28.4 (5.1)	DXA		N/A	N/A	1.08 (1.04–1.12)	Yes
Tanamas, Australia [40]	136 (114 women), 75 pain, 61 no pain	47.5 (9.2) pain 47.7 (8.8) no pain	35.1 (7.8) pain 28.4 (7.6) no pain	DXA	FMI	N/A	N/A	1.16 (1.06–1.28)	Yes
Butterworth, Australia [42]	796 (all men), 177 pain, 619 no pain	68 (24–90)b pain 57 (25–98)b no pain	28.0 (4.3) pain 27.1 (3.8) no pain	DXA		N/A	N/A	1.08 (1.01–1.15)	Yes

*OR* odds ratio, *CI* confidence interval, *BMI* body mass index, *kg* kilogram, *m*^*2*^ metres squared, *DXA* Dual-energy X-ray absorptiometry, *FMI* fat mass index, *BIA* bioelectrical impedance analysis, *N/A* not applicable

^a^Values are mean (SD) unless otherwise stated

^b^median (range)

^c^mean (95% CI)

^d^cross-sectional and longitudinal study

^e^Duplicate study

### Sensitivity analysis

The association between knee pain and body fat percentage was not significant when the data pertaining to women from Scott et al. [39] was removed from the meta-analysis, (SMD 0.16, 95% CI -0.02-0.34, *p* = 0.075), suggesting that the relationship may be mediated by gender. All other sites remained significant when one study was removed from the respective model.

### Publication bias

No significant publication bias was detected for studies reporting foot pain or knee pain, with Egger’s regression intercept (95% CI) of 0.75 (− 2.38–3.87), *p* = 0.412 and − 0.61 (− 10.87–9.66), *p* = 0.589, respectively. There was however a potential for publication bias detected for studies reporting low-back pain with Egger’s regression intercept (95% CI) of 3.44 (1.57–5.33), *p* = 0.004. Widespread pain was reported in only two studies and was therefore not amenable for the funnel plot test or Egger’s regression intercept.

### Cross-sectional studies not included in the meta-analysis

The cross-sectional studies not included in the meta-analysis (due to the type of data or the parameter used) were generally concordant with the overall findings (Table 1), with the reasons for exclusion in Additional file 3. Multi-site pain (3 sites) was associated with FMI in the study by Brady et al. [18]. Neck pain was associated with body fat percentage in one study [45], while temporomandibular pain was not [44]. The large study by Chou et al. [38] that investigated low-back pain used the same sample as reported by Butterworth et al. [42] who investigated foot pain, with both finding FMI, but not FFMI, to be significantly associated with pain. Celan et al. [46] studied the relationship between body fat percentage and low-back pain, but the only data provided were mean body fat percentage, without confidence intervals or standard deviations and therefore these data were not amenable for the meta-analysis. Iizuka et al. [37] investigated multiple regions separately (neck / shoulder, back and low-back) and their associations between body fat percentage. Whilst we felt it appropriate to include the low-back region in the meta-analysis, we did not include the neck / shoulder and the back region with the other studies given the difficulty with delineating these regions, particularly the low-back region from the back region, but we did include the neck / shoulder region in Table 1. Other studies investigating low-back pain generally found increased fat mass was associated with pain. The smaller studies that investigated both knee and shin pain found non-significant associations between pain and body fat mass.

### Longitudinal studies

Findings from the longitudinal studies (Table 2) were consistent with the overall theme identified in the cross-sectional studies, finding increased levels of body fat predicted future musculoskeletal pain. Higher baseline FMI was predictive of foot pain in the short term (less than 3 years) [16, 19] in data from both a community cohort (OR 1.06, 95% CI 1.02–1.11) and a musculoskeletal study (OR 1.28, 95% CI 1.04–1.57). In the knee, Jin et al. [55] found an association between increased fat mass and an increased relative risk (RR) of pain in either lying in bed, (RR 1.47, 95% CI 1.12–1.93) or sitting (RR 1.46, 95% CI 1.10–1.95), although knee pain when weight-bearing was not associated with fat mass. More frequent knee pain at 5.1 years follow-up was positively associated with higher total fat mass, and there was an increased risk (95% CI) of consistent (RR 1.89, 95% CI 1.43–2.51) and fluctuating knee pain (RR 1.78, 95% CI 1.41–2.25). A five-year longitudinal study by Pan et al. [35] found a significant trend across three time-points for fat mass and multisite pain, with the number of painful sites significantly associated with total body fat mass over 5 years. There was, however, some discordance between the relationship of body composition and low-back pain, but the larger studies found fat mass to be a predictor of increased pain and disability following multiple adjustments [56–58]. A twin study by Dario et al. [57] did not find a significant relationship between body fat and the risk of chronic low-back pain in women (*n* = 314), however a larger study (*n* = 4986) by Hussain et al. [56] found higher body fat at baseline to be predictive of both high pain intensity and high disability in women and men at 5 years follow-up. Hashimoto et al. [58] also found that men in the fourth quartile of body fat percentage had a significant risk of chronic back pain at > 20 years follow up when adjusting for age, smoking, alcohol consumption and maximal oxygen uptake (OR 2.12, 95% CI 1.13–3.98). One study found that the risk of developing injury during a three-month training program increased in women with an increased body fat percentage (OR 1.16, 95% CI 1.00–1.34) [54].

**Table 2:** Characteristics of longitudinal articles included in the review (n = 8)^a^

Study / country / reference	Follow-up time	Sample size, n	Age, y	BMI, kg/m^2^ (Baseline)	Body composition assessment	Parameter investigated	Body composition (Baseline)	OR (CI)
Multi-site pain (0–7 joints)								
Pan, Australia [35]^d^	2.6 and 5.1 years	336 widespread pain, 137 no pain	Baseline 63.3 (7.7) widespread pain, 62.2 (7.2) no pain	Baseline 28.8 (5.3) widespread pain, 26.2 (3.9) no pain	DXA	Total fat mass	30.0 (9.5) kg, 25.0 (7.1) kg	1.06 (1.02–1.10)
Incident low-back pain								
Hussain, Australia [56]	5 years	No intensity: 900 Low intensity: 3085 High intensity: 1001 No disability: 3061 Low disability: 651 High disability: 482	49.2 (10.9)	26.6 (4.7)	BIA	Body fat percentage	Pain intensity No: 31.7 (11.8) % Low: 32.6 (11.6) % High 36.2 (13.3) % Pain disability No: 32.1 (11.5) % Low: 33.7 (12.6) % High 37.4 (13.3) %	Men Low intensity: 1.28 (1.09–1.27) High Intensity: 1.45 (1.19–1.77) Low disability: 1.11 (0.92–1.32) High disability: 1.37 (1.10–1.72) Women Low intensity: 1.41 (1.25–1.59) High intensity: 1.39 (1.22–1.57) Low disability: 1.20 (1.07–1.35) High disability: 1.48 (1.31–1.68)
Dario, Spain [57]	4 years	314 (all women)	53.7 (7.0), range 43–71	27.3 (4.0)	BIA		34.1 (7) %	Chronic pain: 0.87 (0.66–1.14) Activity-limiting pain: 0.85 (0.62–1.53) Care-seeking due to pain: 0.79 (0.59–1.05)
Hashimoto, Japan [58]	> 20 years	1152 (all men)	28.0 (4.6)	22.6 (2.7)	Skin fold		14.7 (3.5) %	Q1: referent Q2: 0.86 (0.43–1.71) Q3: 1.46 (0.79–2.72) Q4: 2.12 (1.13–3.98)
Increasing knee pain								
Jin, Australia [55]	5.1 years	767 (380 women)	62.4 (7.2) pain increase 61.9 (7.0) no pain increase	29.1 (5.3) pain increase 27.3 (4.3) no pain increase	DXA	Body fat percentage	Pain increase: 30.2 (7.8) %, no pain increase: 27.0 (7.8) %	1.36 (1.20–1.55)^c^
Incident foot pain								
Butterworth, Australia [19]	3 years	51 (37 women), 11 incident pain, 40 no pain	48.3 (9.8) incident pain, 49.5 (7.9) no pain	29.6 (7.9) incident pain, 26.3 (5.4) no pain	DXA	FMI	12.1 (6.4) kg/m^2^ incident pain, 8.7 (4.2) kg/m^2^ no pain	1.28 (1.04–1.57)
Future foot pain								
Walsh, Australia [16]	4 years	1066	64.6 (10.3)	28.4 (5.1)	DXA	FMI	10.2 (3.9) kg/m^2^	1.06 (1.02–1.11)
Multi-site injuries								
Kodesh, Israel [54]	3 months	158 (all women)	19.0 (18.1–20.2)	20.8 (16.1–32.0)	Skin fold	Body fat percentage^b^	Injured 23.7 (20.5–29.2) % Non injured 22.5 (14.9–31.5) %	1.16 (1.00–1.34)

*OR* odds ratio, *CI* confidence interval, *BMI* body mass index, *kg* kilograms, *m*^*2*^ metres squared, *Q* quartile, *DXA* Dual-energy X-ray absorptiometry, *FMI* fat mass index, *BIA* bioelectrical impedance analysis

^a^Values are mean (SD) unless otherwise stated

^b^median (range)

^c^Relative risk (CI)

^d^cross-sectional and longitudinal study

## Discussion

This is the first review to systematically appraise and synthesise studies examining the relationship between body fat and musculoskeletal pain. This review included single- and multi-site joint pain and the meta-analyses demonstrated significant associations between increased fat mass and widespread pain, low-back pain, knee pain and foot pain. There was also emerging evidence from longitudinal studies that elevated body fat may infer an increased risk of incident or worsening joint pain. Thus, musculoskeletal pain may be a manifestation of excessive fat mass, which exists beyond excessive mechanical loading.

The association between fat mass and widespread pain is perhaps the most important finding of this review. Single-site pain may be confounded by local biomechanical factors or trauma, whereas widespread pain may be due to the pervasive nature of excessive adipose tissue on pain, extending beyond local tissue disease to include how pain may be perceived centrally [59]. The study by Pan et al. [35] found both cross-sectional and longitudinal associations with widespread pain and they adjusted for psychological health in the longitudinal analysis, which is particularly important given the bidirectional relationship between depression and pain [25]. Whilst depressive symptoms are undoubtedly more common in those with excessive adiposity, there were independent associations between body fat and pain, particularly in the foot [16, 40, 42]. The foot is the first site in the body to modulate ground reaction forces, where the bones and soft tissues are subjected to bending and torsional loads [60]. The weak pooled estimate for the association between foot pain and body fat may be attributed to the fact that three of the four articles included in the meta-analysis adjusted for age, gender and depression and normalised fat mass for height, while the other article also matched on age, gender and BMI. This therefore suggests that unless FMI is associated with specific changes to foot mechanics, which seems unlikely, that the association of foot pain with obesity may be metabolically mediated. It is important to note that the magnitude of the effects were small to medium in size, suggesting a relatively modest potential contribution of fat mass to musculoskeletal pain amongst other known physiological and psychological factors.

A number of proposed pathways can explain the association between body fat and musculoskeletal pain, including the up-regulation of cytokines secreted by adipose tissue, referred to as adipokines. Leptin, a pro-inflammatory adipokine predominately expressed by subcutaneous adipose tissue [61] is associated with bodily pain in women [62] and leptin levels in both serum [63] and synovial fluid [64] are associated with osteoarthritis, particularly in women. Leptin has functional receptors on articular chondrocytes, and may be involved with cartilage generation [65]. Leptin signaling, however, may be blunted with adiposity, through a regulative negative feedback loop [66]. Interestingly, excessive adiposity may increase leptin secretion, which in turn may compromise its ability to repair joint cartilage by a down-regulation in receptor expression [67]. This theory is supported by an observational study investigating knee joint changes using magnetic resonance imaging, where reduced cartilage volume, a hallmark of osteoarthritis, is associated with increased leptin [68]. Thus, leptin may be associated with structural joint changes that, at the very least may predispose the joint to further cartilage failure and pain.

Other suggested mechanisms linking adipose tissue with pain, including subclinical inflammation [69, 70]. Tumour necrosis factor-alpha (TNF-α) is a cytokine involved in the inflammatory cascade. It is a therapeutic target for the management of inflammatory arthropathies, and is primarily produced by activated macrophages, but it is also secreted by adipose tissue [17]. Systemic inflammation is up-regulated with obesity with the acute inflammatory phase marker, C-reactive protein (CRP), higher in obese people [71]. The increase in inflammation may be in response to over-nutrition initiating an immune response [72], particularly linked to the consumption of dietary fats [73]. Moreover, elevated TNF-α, along with other inflammatory mediators and markers are associated with chronic pain [74]. Elevated synovial TNF-α levels are also predictive of pain severity and a poor outcome following temporomandibular joint surgery [75]. Furthermore, elevated serum levels of TNF-α and interleukin-6 (IL-6) are associated with less improvement to treatment in those with chronic pain [76] and TNF-α may moderate the relationship between chronic back pain and depressive symptoms [77].

Systemic inflammation related to adiposity has been linked to other structural joint changes and this may be one phenotype that contributes to osteoarthritis [78]. In the knee, both TNF-α and IL-6 have been associated with knee cartilage loss [24] and elevated IL-6 is a predictor of radiographic osteoarthritis [79], suggesting a link between low-level inflammation and osteoarthritis pathogenesis. Tendinopathy has also been linked with dietary fats, adiposity and inflammation [80, 81], highlighting that obesity may not necessarily be only related to excessive load. Clearly elevated body fat is linked with structural changes and pain in multiple regions and may explain the known link between elevated BMI and osteoarthritis in non-weight-bearing joints such as the hands [15]. Future work to investigate if there is a true discordance between fat mass and fat-free mass may help strengthen the notion that body composition is more meaningful measure of risk for musculoskeletal pain.

This review should be considered in light of certain limitations. Firstly, given the lack of homogeneity in follow-up time, we were unable to undertake a meta-analysis on longitudinal associations between musculoskeletal pain and body fat. Secondly, despite the considerable variability in the quality of the articles included in this study, a number of items assessed with the EAI would have scored higher had they been explicitly reported, such as the reliability and validity of the tools used to assess pain and body composition. A number of the tools are known to be both reliable and valid, but unfortunately this was not reported by the authors. Thirdly, the case-definition for pain did vary between studies and thus while we did perform a meta-analysis by region those with stricter criteria may under-report the prevalence, incidence or progression of pain. Fourthly, the pooled estimates of the meta-analyses are small to medium in size, suggesting a weak to moderate effect which should be taken into consideration. Finally, this review focused on the association between body fat and pain, but it did not investigate whether lean mass was inversely related to pain. However, this is the first review to systematically appraise and synthesise studies examining the relationship between body fat and musculoskeletal pain.

## Conclusion

This systematic review has demonstrated that increased body fat is positively associated with widespread pain, low-back pain, knee pain and foot pain. Meta-analysis found positive cross-sectional associations between increased body fat and widespread and single-site joint pain in the low-back, knee and foot. Evidence from longitudinal studies suggests elevated body fat may infer increased risk of incident and worsening joint pain, although further high-quality studies are required.

## Additional files

Additional file 1: Ovid Medline search strategy. Example of database search strategy. (DOCX 73 kb)

Additional file 2: Quality assessment. Quality assessment of the articles included in the systematic review. (DOCX 140 kb)

Additional file 3: Reasons for exclusion from meta-analysis. Reasons for exclusion of cross-sectional studies from the meta-analyses. (DOCX 135 kb)

## Abbreviations

BIA: Bioelectrical impedance analysis
BMI: Body mass index
CI: Confidence interval
cm: Centimetre
CRP: C-reactive protein
DXA: Dual-energy X-ray absorptiometry
EAI: Epidemiology Appraisal Instrument
FFMI: Fat-free mass index
FMI: Fat mass index
IL-6: Interleukin-6
NJ: New Jersey
OR: Odds ratio
PRISMA: Preferred Reporting Items for Systematic Reviews and Meta-Analyses
RR: Relative risk
SMD: Standardised mean differences
TNF-α: Tumour necrosis factor-alpha
USA: United States of America
