# Supplementary material for: The association between body fat and musculoskeletal pain: a systematic review and meta-analysis
Source: BMC Musculoskelet Disord. 2018 Jul 18;19:233. doi: 10.1186/s12891-018-2137-0 (PMC6052598; doi:10.1186/s12891-018-2137-0)
Supplement: Supplementary file 1 — Ovid Medline search strategy. Example of database search strategy. (DOCX 73 kb) [file 12891_2018_2137_MOESM3_ESM.docx]

Additional file 3: Reasons for exclusion from meta-analysis

| **Study** | **Reason for exclusion** |
| --- | --- |
| Brady [18] | This study did not stratify each body site by body composition and it was the only study to report up to three body sites. |
| Celan [46] | Unable to calculate effect size or odds ratio from the data provided |
| Chou [38] | The other studies assessing low-back pain measured body fat %, rather than fat mass or fat mass index |
| Iizuka [37] | Only study to report neck and shoulder pain |
| Jordani [44] | The participants with and without pain were compared according to their body fat %, but were stratified into four categories |
| Sabeti [50] | Only study to report shin pain |
| Sutbeyaz [53] | The other studies assessing knee pain measured body fat %, rather than fat mass or fat mass index |
| Urquhart [20] | Pain reported is not binary and is stratified into disability and intensity, unlike the other studies that reported low-back pain |
| Yalcinkaya [45] | Only study to report neck pain |
