# Supplementary material for: The association between body fat and musculoskeletal pain: a systematic review and meta-analysis
Source: BMC Musculoskelet Disord. 2018 Jul 18;19:233. doi: 10.1186/s12891-018-2137-0 (PMC6052598; doi:10.1186/s12891-018-2137-0)
Supplement: Supplementary file 2 — Quality assessment. Quality assessment of the articles included in the systematic review. (DOCX 140 kb) [file 12891_2018_2137_MOESM2_ESM.docx]

Additional file 2: Quality assessment

| **Study** | **1. Hypothesis/aim/objective** | **2.Exposure** | **3. Outcome** | **4. Study design** | **5.Study population** | **6. Inclusion criteria clearly described** | **7.Participation rates reported** | **8.Characteristics of participants described** | **9.Characteristics of subjects lost or unavailable reported** | **11. Important covariates described** | **13.Statistical methods described** | **14.Results clearly described** | **15.Variability in data reported** | **16.Statistical parameters reported** | **17. Sample size calculation** | **18.Comparability of groups** | **19. Adequate participation rate** | **21.Drop-out characteristics reported*** | **25.Exposure variables reliable** | **26.Exposure variables valid** | **27.Exposure methods comparable** | **28. Exposure conducted prior to symptoms** | **29.Observers blind to subject grouping** | **30.Subjects blinded** | **31.Outcome measures reliable** | **32.Outcome measures valid** | **33.Standardised assessment of variables** | **34.Same time period of observations** | **36. Adequate adjustment for covariates** | **38. Minimum time to follow-up to detect relationship** | **39. Adjustment for different lengths of follow-up** | **41. Exposure data reported by subgroup** | **42.Generalisability of results to study population** | **43.Generalisability of results to greater population** | **Summary of questions answered ‘yes’ (%)** |
| --- | --- | --- | --- | --- | --- | --- | --- | --- | --- | --- | --- | --- | --- | --- | --- | --- | --- | --- | --- | --- | --- | --- | --- | --- | --- | --- | --- | --- | --- | --- | --- | --- | --- | --- | --- |
| Brady [18] | y | y | y | p | p | p | n | y | n | y | p | y | y | n | n | na | utd | n | utd | utd | na | na | na | na | utd | utd | na | na | y | na | na | y | utd | utd | 36 |
| Butterworth [19] | y | y | y | p | p | p | y | y | y | y | y | y | y | y | n | y | utd | p | utd | utd | y | y | utd | utd | utd | y | y | utd | y | y | utd | y | y | y | 62 |
| Butterworth [42] | y | y | y | y | y | n | y | y | y | y | y | y | y | y | n | na | y | y | utd | utd | na | na | na | na | utd | y | na | na | y | na | na | y | y | y | 80 |
| Celan [46] | n | p | p | y | p | p | y | n | n | n | p | n | n | n | n | y | utd | n | utd | utd | y | y | na | utd | utd | utd | y | utd | n | na | na | y | utd | utd | 23 |
| Chou [38] | y | y | y | y | y | p | y | y | p | y | y | y | y | y | n | na | y | p | utd | utd | na | na | utd | utd | utd | y | na | na | y | na | na | y | y | y | 67 |
| Dario [47] | y | y | y | y | y | p | n | y | n | n | p | y | y | y | n | na | utd | n | utd | utd | na | na | utd | utd | utd | utd | na | na | n | na | na | y | utd | utd | 37 |
| Dario [57] | y | y | y | y | y | n | y | y | y | p | y | y | y | y | n | na | utd | y | utd | utd | na | y | na | na | utd | utd | na | na | p | y | y | y | y | y | 68 |
| Hashimoto [58] | y | y | y | p | y | n | y | n | n | n | y | p | y | y | n | y | utd | utd | utd | utd | y | n | utd | utd | utd | utd | y | y | p | y | y | n | p | n | 41 |
| Hodselmans [51] | y | y | p | y | y | n | n | y | n | n | y | y | y | n | n | p | utd | utd | utd | utd | utd | na | n | n | utd | utd | utd | utd | n | na | na | p | utd | utd | 26 |
| Hussain [56] | y | y | y | y | y | n | y | y | y | y | y | y | y | y | n | y | y | y | utd | utd | y | y | y | y | y | y | y | y | y | y | utd | y | y | y | 85 |
| Iizuka [37] | y | y | y | y | y | p | y | y | n | p | y | y | y | y | n | na | n | y | utd | utd | na | na | na | na | utd | utd | na | na | p | na | na | n | n | n | 48 |
| Jin [55] | y | y | y | y | y | p | y | y | n | p | y | y | y | y | n | y | p | y | utd | utd | y | y | utd | y | utd | y | y | y | p | y | utd | y | p | p | 62 |
| Jordani [44] | y | y | p | n | y | y | p | y | n | y | y | y | y | y | n | y | utd | y | utd | utd | y | na | utd | utd | utd | utd | y | p | p | na | na | y | utd | utd | 48 |
| Kodesh [54] | y | y | p | p | y | p | n | y | n | n | y | y | y | y | n | y | utd | n | y | utd | y | y | y | y | utd | utd | y | y | n | y | na | y | utd | utd | 55 |
| Ozer Kaya [49] | y | y | y | y | y | p | y | y | n | n | y | y | y | n | n | y | p | n | utd | utd | y | na | utd | utd | y | p | y | utd | n | na | na | y | p | p | 48 |
| Pan [35] | y | y | y | y | y | n | n | y | n | y | y | y | y | y | n | y | utd | p | utd | utd | y | utd | utd | utd | utd | utd | y | y | y | y | y | y | utd | utd | 56 |
| Sabeti [50] | y | y | y | p | n | p | n | y | n | n | p | p | p | n | n | utd | utd | y | utd | utd | y | na | n | utd | utd | utd | y | utd | n | na | na | y | p | utd | 37 |
| **Study** | **1. Hypothesis/aim/objective** | **2.Exposure** | **3. Outcome** | **4. Study design** | **5.Study population** | **6. Inclusion criteria clearly described** | **7.Participation rates reported** | **8.Characteristics of participants described** | **9.Characteristics of subjects lost or unavailable reported** | **11. Important covariates described** | **13.Statistical methods described** | **14.Results clearly described** | **15.Variability in data reported** | **16.Statistical parameters reported** | **17. Sample size calculation** | **18.Comparability of groups** | **19. Adequate participation rate** | **21.Drop-out characteristics reported*** | **25.Exposure variables reliable** | **26.Exposure variables valid** | **27.Exposure methods comparable** | **28. Exposure conducted prior to symptoms** | **29.Observers blind to subject grouping** | **30.Subjects blinded** | **31.Outcome measures reliable** | **32.Outcome measures valid** | **33.Standardised assessment of variables** | **34.Same time period of observations** | **36. Adequate adjustment for covariates** | **38. Minimum time to follow-up to detect relationship** | **39. Adjustment for different lengths of follow-up** | **41. Exposure data reported by subgroup** | **42.Generalisability of results to study population** | **43.Generalisability of results to greater population** | **Summary of questions answered ‘yes’ (%)** |
| Sakai [43] | y | y | y | y | y | p | n | y | n | p | y | y | y | n | n | y | utd | n | utd | utd | y | na | utd | utd | utd | utd | y | y | n | na | na | y | utd | utd | 45 |
| Scott [39] | y | y | y | y | y | p | p | y | n | n | y | y | y | y | n | y | p | n | utd | utd | y | na | utd | utd | utd | y | y | y | n | na | na | y | p | p | 52 |
| Spyropoulos [52] | y | y | y | n | y | p | n | y | n | n | p | y | y | n | n | y | utd | n | utd | utd | y | na | y | n | utd | utd | y | y | n | na | na | y | utd | utd | 42 |
| Sutbeyaz [53] | y | y | y | p | y | y | n | y | n | p | y | y | y | n | n | y | utd | n | utd | utd | y | na | utd | utd | utd | y | y | utd | n | na | na | y | utd | utd | 45 |
| Tanamas [40] | y | y | y | p | p | p | n | y | n | y | y | y | y | y | n | na | utd | n | utd | utd | na | na | na | na | utd | y | na | na | y | na | na | y | utd | utd | 48 |
| Toda [48] | p | y | y | p | p | p | n | y | n | p | y | y | y | n | n | y | utd | n | utd | y | y | na | utd | utd | utd | utd | y | y | n | na | na | y | utd | utd | 39 |
| Urquhart [20] | y | y | y | y | p | p | n | y | n | y | n | y | y | y | n | na | utd | n | utd | utd | na | na | na | na | y | y | na | na | p | na | na | y | utd | utd | 48 |
| Walsh [16] | y | y | y | p | y | n | n | y | n | y | y | y | y | y | n | y | utd | n | utd | utd | y | y | utd | utd | utd | utd | y | y | y | y | utd | y | utd | utd | 53 |
| Walsh [41] | y | y | y | y | y | y | n | y | n | y | y | y | y | y | y | p | utd | n | y | utd | y | na | n | n | y | p | y | utd | p | na | na | y | utd | utd | 58 |
| Yalcinkaya [45] | y | y | y | p | y | y | n | y | n | y | y | y | y | n | n | p | utd | n | utd | utd | y | na | n | n | y | y | y | utd | n | na | na | y | utd | utd | 48 |
| Yoo [36] | y | y | y | y | y | y | y | y | y | p | y | y | y | y | n | y | p | n | utd | utd | y | na | na | na | utd | utd | y | na | p | na | na | y | p | p | 61 |
| **Studies scoring 'yes' (%)** | 93 | 96 | 86 | 57 | 75 | 18 | 39 | 93 | 18 | 43 | 79 | 89 | 93 | 64 | 4 | 57 | 11 | 25 | 7 | 4 | 68 | 25 | 11 | 11 | 18 | 37 | 68 | 36 | 29 | 29 | 11 | 89 | 18 | 18 |  |

*y* conditions met, *p* conditions partially met, *n* conditions not met, *utd* unable to determine, *na* not applicable
