# Supplementary material for: The association between body fat and musculoskeletal pain: a systematic review and meta-analysis
Source: BMC Musculoskelet Disord. 2018 Jul 18;19:233. doi: 10.1186/s12891-018-2137-0 (PMC6052598; doi:10.1186/s12891-018-2137-0)
Supplement: Supplementary file 3 — Reasons for exclusion from meta-analysis. Reasons for exclusion of cross-sectional studies from the meta-analyses. (DOCX 135 kb) [file 12891_2018_2137_MOESM1_ESM.docx]

Additional file 1: Ovid Medline search strategy

| # | Searches |
| --- | --- |
| 1 | adipocytes/ or adipose tissue/ or adipose tissue, beige/ or adipose tissue, brown/ or adipose tissue, white/ or abdominal fat/ or intra-abdominal fat/ or subcutaneous fat, abdominal/ or subcutaneous fat/ |
| 2 | Anthropometry/ or body composition/ or body fat distribution/ or adiposity/ |
| 3 | obesity, abdominal/ |
| 4 | (body adj4 composition).tw,kw. |
| 5 | (Anthropometr* or "Lean to fat").tw,kw. |
| 6 | (Adipos* or adipocyte*).tw,kw. |
| 7 | ((Body or trunk or subcutaneous or visceral or abdominal or android or gynoid) adj3 fat).tw,kw. |
| 8 | (fat adj (mass or deposit* or content or accumulat* or muscle or tissue or volume* or percentage or distribut* or thickness or ratio?)).tw,kw. |
| 9 | ((Trunk or subcutaneous or visceral or abdominal or android or gynoid) adj obesity).tw,kw. |
| 10 | or/1-9 |
| 11 | musculoskeletal pain/ or myalgia/ or fibromyalgia/ or arthralgia/ |
| 12 | shoulder pain/ or back pain/ or low back pain/ or pelvic girdle pain/ or neck pain/ |
| 13 | Muscles, skeletal/ or Joints/ or tendons/ or ligaments/ or ligaments, articular/ or "bone and bones"/ or exp cartilage/ |
| 14 | hip/ or hip joint/ or knee/ or knee joint/ or foot/ or heel/ or leg/ or lower extremity/ |
| 15 | upper extremity/ or arm/ or elbow/ or forearm/ or hand/ or shoulder/ or neck/ or back/ or lumbosacral region/ or sacrococcygeal region/ |
| 16 | "bones of lower extremity"/ or "bones of upper extremity"/ or spine/ |
| 17 | cervical vertebrae/ or coccyx/ or intervertebral disc/ or lumbar vertebrae/ or sacrum/ or thoracic vertebrae/ |
| 18 | pain/ or acute pain/ or breakthrough pain/ or chronic pain/ or metatarsalgia/ or morton neuroma/ or exp neuralgia/ or pain, intractable/ |
| 19 | Pain Measurement/ |
| 20 | or/13-17 |
| 21 | or/18-19 |
| 22 | 20 and 21 |
| 23 | ((muscular or muscle* or joint* or musculo* or bone* or skeletal or tendon* or ligament* or cartilage) adj3 pain).tw,kw. |
| 24 | (myalgi* or fibromyalgi* or arthralgi* or metatarsalgi*).tw,kw. |
| 25 | (((shouder* or back or pelvic or spine or spinal or neck or vertebrae or vertebral or intervertebral or arm* or hand* or elbow* or forearm* or upper extremit* or limb* or widespread) adj3 pain*) or backache).tw,kw. |
| 26 | ((hip or hips or knee* or foot or feet or heel or heels or leg or legs or lower extremit*) adj3 pain*).tw,kw. |
| # | Searches |
| 27 | or/23-26 |
| 28 | 11 or 12 or 22 or 27 |
| 29 | 10 and 28 |
| 30 | exp animals/ not humans/ |
| 31 | (mice or mouse or murine or rat or rats or rabbit* or equine or horse*).ti. |
| 32 | (case reports or comment or editorial or legal cases or legislation or letter or news or newspaper article or patient education handout).pt. |
| 33 | or/30-32 |
| 34 | 29 not 33 |
| 35 | limit 34 to english language |

Notes: / = Medical Subject Heading (MeSH) search; tw = search on title and abstract fields; kw = search on author keywords; ti = search on title field only; pt = publication type search; exp = exploded MeSH term search to include narrower headings; adj = adjacency operator, restricting search terms on either side to occur within a designated number of spaces from each other.
